# An intervention of sustainable weight change: Influence of self‐help group and expectations

**DOI:** 10.1111/hex.13290

**Published:** 2021-06-05

**Authors:** Kristina Carlén, Elisabeth Kylberg

**Affiliations:** ^1^ School of Health Sciences University of Skövde Skövde Sweden

**Keywords:** behavioural change, expectation, obesity, physical activity, self‐help group, weight change

## Abstract

**Background:**

Obesity is one of the most challenging public health problems in Western societies. Group activities are a way to empower individuals to make sustainable lifestyle changes. Self‐help groups enable individuals to share expectations and experiences on an equal basis.

**Objective:**

The aim was to find a model for sustainable weight reduction for people with obesity and to evaluate the importance of expectations before entering the weight reduction programme.

**Methods:**

Persons with a BMI >30 and aged over 30 years were recruited. Weekly seminars for 6 months with discussions concerning physical activity, eating habits and how to change one's lifestyle occurred. After the seminars, a self‐help group was initiated. The participants were encouraged to express their expectations before each step in the study.

**Results:**

Our findings showed that those who had joined a self‐help group had reduced their weight significantly (−6.0 kg) compared with those who had not (−1.4 kg). Further, those who expressed a more mature expectation of the coming change in behaviour towards a healthy lifestyle showed slightly larger weight reduction (−6.1 kg) than those who expressed low expectations (−3.7 kg).

**Patient or public contribution:**

Participants expressed their thoughts and views, which were considered and included in the programme.

**Conclusions:**

Our findings indicate that the self‐help group can be an essential part of a weight reduction programme. The self‐help group is a novel strategy to strengthen sustainability in reducing weight. The study also highlights the importance of identifying behaviour change expectations before participating in a programme.

## 1 INTRODUCTION

### 1.1 Obesity

Obesity is one of the most challenging public health problems in Western societies. Worldwide, obesity has nearly tripled since 1975.^1^ In North and South America, Europe and the Middle East, approximately half of the population is overweight or obese. At the same time, a lower prevalence of 14%‐27% is observed in Africa, South‐East Asia and Western Pacific.^2^, ^3^ In the Nordic population, there has been an estimated increase in obesity of 1.4 percentage points between 2011 and 2014.^4^ Furthermore, between 2004 and 2017, the percentage of obese people in Sweden increased from 11% to 15% between 16 and 84 years of age.^5^ The increasing prevalence of obesity is related to changes in lifestyle.^1^ Obesity is associated with a higher risk of morbidity caused by metabolic syndrome and reduced well‐being, mentally as well as psychosocially.^6^ Few weight‐reduction programmes have had a design with the long‐time follow‐up, enabling us to evaluate whether the weight loss is sustainable. Existing data suggest that weight regain is a frequent problem and begins 12‐24 months after weight reduction.^7^

### 1.2 Obesity is preventable

One of the most apparent causes of overweight and obesity, the imbalance of calories consumed and calories expended, is widely understood. The consumption of fat and sugar has increased globally perspective, while the amount of physical activity has decreased. The reduction in physical activity has occurred due to the nature of many types of jobs, new transportation methods and increasing urbanization.^1^ Obesity is preventable, on a public health level, through political decisions regarding economic consequences from a global health perspective.^8^

### 1.3 Weight lost motivations and expectations

Relatively small reductions in weight have clinically relevant benefits, but long‐term weight‐loss maintenance (WLM) is challenging.^9^ Behaviour change interventions are identified as keys for WLM, where the motivation factor is essential. Motivation is crucial to support behaviour change, and motivational interviewing (MI) has been identified as a successful approach to behaviour change for health benefits.^10^ MI is a counselling method that facilitates finding an internal motivation to change behaviour by helping clients to explore and resolve ambivalence.^11^ A review concerning long‐term weight loss with a multidisciplinary approach stated that a high motivation factor is essential for long‐term weight management^12^ and behaviour areas like physical activities, psychological and biological components, and cognitive components affect the duration WLM. Factors influencing behaviour change were identified, including motivation, the ability to set small and achievable goals, and the programme's expectation.^13^

The transtheoretical model (TTM) of health behaviour change^14^ describes a process that takes place through six stages of change: pre‐contemplation, contemplation, preparation, action, maintenance and termination. Behaviour change is often associated with, for example, quitting smoking or drinking. However, behavioural change is essential to finding a new lifestyle regarding weight loss and maintaining the new weight. Pre‐contemplation is the stage where the individual does not yet even intend to act, at least not within the next 6 months. Contemplation is the stage where the individual intends to act within the next 6 months. There is a balance between costs and benefits, which causes ambivalence in the person. Preparation is the stage in which the individual intends to act within the next month. The individual usually has a plan for action, such as taking part in an intervention programme. Action is the stage where the individual has made the behaviour change. Maintenance is the stage where the individual is working to prevent a relapse. Relapse is not a separate stage; it is a form of regression. Termination is the stage in which the individual has total control (self‐efficacy). The individual perceives that she or he does not want to return to the old habit. This model can constitute an essential guide for intervention programmes.^14^ However, Bandura's Social Learning Theory^15^ attempts to predict and explain behaviour using several key concepts: incentives, outcome expectations and self‐efficacy expectations.^16^ Although all are important, the concept of self‐efficacy expectations is of relevance to health education. Bandura's assertion that efficacy expectations reflect a person's perceived, rather than actual, capabilities, and these perceptions and not one's true abilities often influence behaviour.

### 1.4 Self‐help group

A self‐help group is a group in which members provide each other with various support types for a special identified and shared characteristic.^17^ The self‐help group is voluntary and based on mutual equality and reciprocity. There are four dimensions within a self‐help group; the shared understanding, mutual assistance, the possibility of obtaining information and the social community built on a social cohabitation. The sharing of experiences among the participants contributes to a common understanding within the group. For the individual as well as the group, this understanding creates meaning and constitutes the experience‐based knowledge.^18^ Group dynamics shows to influence weight loss in an intervention programme. When there was a conflict in a group, the participants had smaller weight reduction.^19^ In a study among older adults, researchers found that group interventions for weight reduction must focus on social activities and health identity instead of a weight identity.^20^ Reflection concerning the way of living related to the emotional balance, as one part of the support in a group, contributed to sustainable weight reduction. The weight change was more considerable if the individual focused on health and well‐being instead of an identity focus determined by weight. The group is a resource for lifestyle changes, including establishing psychological connections with other persons or sharing a social identity.^21^ However, one crucial part of social support to gain sustainable lifestyle changes was to share personal challenges and experiences. A self‐help group is based on respect for each other, the fact that everyone participates voluntarily, and the act of active listening.^17^

There is a need for effective programmes to promote healthy behaviour for improving public health.^22^ The current study aimed to find a model for sustainable weight reduction for people with obesity and evaluate the importance of expectations before entering the weight reduction programme.

## 2 METHODS

The study was a prospective study with a longitudinal follow‐up. The Regional Ethical Review Board in Gothenburg, Sweden, approved the study. It was designed and completed in line with Recommendations for the Protection of Research Participants and adhered to the Helsinki Declaration. Written informed consent was obtained from all individual participants included in the study, and they could interrupt their participation at any time.

### 2.1 Recruitment and sample

The participants were recruited through leaflets posted at primary health‐care centres, training facilities and at the University in the region. To be included in the study, the persons must have a BMI >30 kg/m^2^ and be aged 30 years or older. Exclusion criteria were pregnancy and current eating disorders. In total, 30 participants were recruited during 2011‐2013; and 26 of those continued after the first meeting. All participants wanted to reduce weight. Many of the participants had tried different methods to lose weight and had the experience of losing weight and gaining weight again. The participants had different habits according to physical activity. Seventeen participants completed the study with seminars and self‐help groups, and nine participated only in the seminars.

### 2.2 Change behaviour towards a healthy lifestyle

The programme had the intention to motivate and inspire the participants to find their way to change their lifestyle in terms of physical activity and diet, which should result in a sustainable lifestyle. During all the programme stages, participants expressed their thoughts and views, which were considered and included continuously. In order to give the participants the necessary tools, the programme started with seminar series, including 12 themes, once per week. The participants were divided into groups of 8‐12 persons. During these 12 weeks with the seminars, the participants offered to participate in a gym's physical activities. The seminars are described in detail^23^ and included themes such as physical activity, a diet with the emphasis of a regular healthy diet, size of portions, motivation, to set realistic goals in a short perspective as well as in a long perspective, how to solve problems, stress, support, communication, hunger, cravings and how to find a sustainable healthy lifestyle. After the seminars, a self‐help group started. The activity in the self‐help group was based on therapeutic communication methods, which included active listening, reciprocity and respect for each and everyone's thoughts. The participants raised themes for each meeting. The participants expressed their thoughts, feelings about the theme. Each participant was given the opportunity to speak about their own thoughts’ experiences, using the first person, ‘I, me, my’, and not ‘you, they’ until they were ready to pass on the opportunity to the next person. No one commented, valued or interrupted the person talking. The way to communicate in a self‐help group can help raise awareness of why ‘I have my lifestyle and what is necessary to change and how’.^18^ The self‐help group lasted for at least 6 months with a weekly meeting. The self‐help group made it possible to facilitate the sustainability of a changed lifestyle.

### 2.3 Data collection

The weight (excluding shoes) was taken with the same bathroom scale at weekly sessions, seminars and self‐help groups. The participants initially reported background determinants and answered the question about their expectations of participating in the programme. This question was repeated before starting the self‐help group, then after each completed six‐month period in the self‐help group. Three participants were involved in the writing process concerning priority and selected essential findings.

### 2.4 Analysis

The hypothesis, that those who participated in a self‐help group were motivated (empowered) to reduce considerably more weight than those who participated only in the seminars, was tested by *t* test. The expectations were coded and graded on a scale from one to four, where one was regarded as a low expectation, ‘low’, and four was regarded as a mature expectation, ‘mature’. The outcome variable was the weight change from the start and during the attending period. A *t* test was performed to see whether there was a difference between those with more mature expectations than those with low expectations.

## 3 RESULTS

Seventeen women and nine men participated in the programme. A total of seventeen participated in the seminars and the self‐help group, and nine participated only in the seminars. The mean age of the participants was 52 years (range 33‐72). The BMI varied between 30.0 and 43.8 kg/m^2^ (Mean 36.1 kg/m^2^) when entering the project. Education, current living conditions and work situation can be seen in Table 1. Fifteen participants (58%) perceived their health as good or very good, and six (23%) as bad or very bad. When estimating their health concerning other individuals, three participants (12%) perceived their health as good or very good, and twelve (47%) as bad or very bad (Table 1).

**TABLE 1:** Characteristics of study participants

Parameter	N = 26
Age (years) Mean (min‐max)	52 (33‐72)
BMI (kg/m^2^) (Mean (min‐max)	36.1 (30.0‐43.8)
Gender, % (n)	
Women	65 (17)
Men	35 (9)
Education, % (n)	
Basic school (9 y)	12 (3)
Gymnasium (12 y)	58 (15)
High school (>12 y)	30 (8)
Living situation, % (n)	
Alone	27 (7)
Family	73 (19)
Current work situation, % (n)	
Full‐time	35 (9)
Part‐time	30 (8)
No work	12 (3)
Pension	23 (6)
Perceived health – my own, % (n)	
Good or very good	58 (15)
Nor/neither	19 (5)
Bad	23 (6)
Very bad	0 (0)
Perceived health – my own in relation to others, % (n)	
Good or very good	12 (3)
Nor/neither	41 (11)
Bad	35 (9)
Very bad	12 (3)

Those participants who participated in the seminars and the self‐help group (n = 17) showed a significantly higher weight reduction, 6.0 kg (Mn) (Min 1.6‐Max −23.8), compared with those who just participated in the seminars (n = 9), 1.4 kg (Mn) (Min 0.7‐Max −4.5) (*P* = .05). Taking part in the self‐help groups varied between 6 and 63 months (Mean 26 months, Median 12 months).

The participants with expectations classified as ‘mature’ expressed a motivation to make a behavioural change and had the ambition to change his/her daily food habits and physical activities. They could also express how to handle their life situation but expressed a need for support from the group. The participants with expectations classified as low expressed a desire to be a part of the programme and have a passive role. They relied on other participants or the leaders, who should support the necessary changes and support social friendship. Those who expressed a more mature expectation of the coming programme (n = 12) showed a higher, but not significantly, weight change, 6.1 kg (Mn) (Min 0.7‐Max −23.8) than those with low expectation (n = 10), 3.7 kg (Mn) (Min 1.6‐Max −13.6) (*P* = .35).

## 4 DISCUSSION

### 4.1 Self‐help group

Those who participated both in the seminars and the self‐help group showed a higher weight reduction than those who only took part in the seminar. The self‐help group helped the participants to adjust to and handle changes in lifestyle associated with improved diet and physical activity. The self‐help group also facilitated the process for those who had a long way to go to find their way to change their lifestyles, initially to find it and establish a new lifestyle concerning food habits and physical activity. The role of self‐help groups connected with weight loss programmes as a key to reducing weight in a long‐term perspective is rarely studied. However, the self‐help group was beneficial for those who had experienced repeated weight‐loss efforts earlier.^24^ The process taking place in a self‐help group describes as a neuropsychological process and an empowerment process.^25^

### 4.2 The role of expectations and motivation

Our results showed that an individual's expectations when entering the project had an impact on weight change. Those with mature expectations had a more considerable weight reduction than those with low expectations. Thus, the expectations may influence the motivation and according to the TTM.^14^ An individual who has started a change process will move between the different stages depending on their motivation. A review stated that the TTM was often used in connection with behaviour change regarding lifestyle.^26^ Recent research in a Spanish context showed the importance of TTM in relation to designing intervention concerning weight reduction.^27^ Bandura declared that expectations of personal efficacy determine whether coping behaviour will initiate or not. The individual experience, performance accomplishments, verbal persuasion and physiological states will influence personal efficacy expectations.^15^ In the expectancy‐value theory, the connection between expectancy and motivation is described. If expecting something as a child, you will be motivated to learn about that or change something in a specific direction.^28^ In our study, the participants perceived their health considerably better than the perception of their health compared to others. This indicates the importance of being clear of the meaning of health perception when asking individuals to estimate their health. From the individual's perspective, a person with a realistic programme expectation has easier to facilitate a behavioural change and maintain weight loss.^13^ If motivation is low and you still are in the stage of pre‐contemplation and must manage problems in that stage, you must thoroughly manage with this before you can proceed into the following stages. Only then can an individual make a sustainable lifestyle change by setting up reasonable and achievable goals and find his or her way. In the self‐help group, you handle experience‐based knowledge^18^ from self and others in the group. This will motivate a change to a more sustainable lifestyle, to find a balance in food intake and physical activities.

Another aspect that can strengthen the efficacy of an intervention programme is to identify encouragers within the individual's network.^29^ Encouragers can also be found within the group. Also, individuals with higher autonomous motivation were less likely to drop out early, which can improve the intervention programme's effectiveness.^30^ The autonomous motivation showed an improvement after 4 months of intervention, confirming that time is needed to change behaviour regarding lifestyle.^31^ When designing a programme for behaviour change concerning weight reduction, it is essential to ask for expectations before attending the programme. The time has come for the health‐care systems to take an active role in promoting public health,^22^ and our model could be one of these activities.

One limitation was the low number of participants in the study, making it impossible to generalize the result. However, the result showed a direction on the expectations regarding the weight differences among the participants in the seminar part compared to the self‐help group. The duration of the participation in the self‐help group showed a considerable variation, and it could influence the sustainability of participants’ weight reduction. The need for support from the group probably made some participants continue the contact with the group. They found the group essential for their daily living. Some of the participants joined the programme due to free training at the gym for 6 months and were not interested in the comprehensive study.

One strength was the duration of participation in the programme; for some persons, up to 5 years, which confirms that the possibility to participate long enough is an important key for sustainable weight reduction. The model seemed to be a cost‐effective programme as the need for professional support was only at the beginning of the study during the seminars; in the latter part, the participants supported each other. According to the WHO’s Sustainable Development Goal (SDG) number three concerning the ability to create health for all, this model could promote public health concerning the prevention of overweight, even for those who cannot afford to take part in expensive programmes for weight reduction.

Our findings indicate that the self‐help group can be an essential part of a weight reduction programme. The self‐help group is a novel strategy to strengthen sustainability in reducing weight. The study also alerts the importance of finding out the expectations before participating in a programme.

## CONFLICT OF INTEREST

The authors declare that they have no conflict of interest.

## Data Availability

The data that support the findings of this study are available on request from the corresponding author.
